# Lentil adaptation to drought stress: response, tolerance, and breeding approaches

**DOI:** 10.3389/fpls.2024.1403922

**Published:** 2024-08-20

**Authors:** Md. Mahmud Al Noor, Md. Tahjib-Ul-Arif, S. M. Abdul Alim, Md. Mohimenul Islam, Md. Toufiq Hasan, Md. Ali Babar, Mohammad Anwar Hossain, Zilhas Ahmed Jewel, Yoshiyuki Murata, Mohammad Golam Mostofa

**Affiliations:** ^1^ Plant Breeding Division, Bangladesh Institute of Nuclear Agriculture, Mymensingh, Bangladesh; ^2^ Department of Biochemistry and Molecular Biology, Bangladesh Agricultural University, Mymensingh, Bangladesh; ^3^ Graduate School of Environmental, Life, Natural Science and Technology, Okayama University, Okayama, Japan; ^4^ Horticulture Division, Bangladesh Institute of Nuclear Agriculture, Mymensingh, Bangladesh; ^5^ Department of Biotechnology, Bangladesh Agricultural University, Mymensingh, Bangladesh; ^6^ Agronomy Departments, University of Florida, Gainesville, FL, United States; ^7^ Department of Genetics and Plant Breeding, Bangladesh Agricultural University, Mymensingh, Bangladesh; ^8^ Faculty of Agriculture, Bangabandhu Sheikh Mujibur Rahman Science and Technology University, Gopalganj, Bangladesh; ^9^ Department of Biochemistry and Molecular Biology, Michigan State University, East Lansing, MI, United States; ^10^ Department of Energy Plant Research Laboratory, Michigan State University, East Lansing, MI, United States

**Keywords:** abiotic stress, morphology, pulse crop, plant growth, omics, water-deficit

## Abstract

Lentil *(Lens culinaris* Medik.) is a cool season legume crop that plays vital roles in food and nutritional security, mostly in the least developed countries. Lentil is often cultivated in dry and semi-dry regions, where the primary abiotic factor is drought, which negatively impacts lentil growth and development, resulting in a reduction of yield. To withstand drought-induced multiple negative effects, lentil plants evolved a variety of adaptation strategies that can be classified within three broad categories of drought tolerance mechanisms (i.e., escape, avoidance, and tolerance). Lentil adapts to drought by the modulation of various traits in the root system, leaf architecture, canopy structure, branching, anatomical features, and flowering process. Furthermore, the activation of certain defensive biochemical pathways as well as the regulation of gene functions contributes to lentil drought tolerance. Plant breeders typically employ conventional and mutational breeding approaches to develop lentil varieties that can withstand drought effects; however, little progress has been made in developing drought-tolerant lentil varieties using genomics-assisted technologies. This review highlights the current understanding of morpho-physiological, biochemical, and molecular mechanisms of lentil adaptation to drought stress. We also discuss the potential application of omics-assisted breeding approaches to develop lentil varieties with superior drought tolerance traits.

## 1 Introduction

Lentil (*Lens culinaris* Medik), one of the most widely grown legumes worldwide, is cultivated on 4.34 million hectares of land and produces 4.95 million tons of pulses annually (Dikshit et al., 2015). The major lentil growing areas in the world encounter various biotic and abiotic stresses, which limit plant growth and ultimately restrict pod yield. Among various abiotic stresses, drought is a major obstacle in lentil cultivation, especially in rainfed ecosystems (Farooq et al., 2017). Drought severely limits the productivity of cool-season legumes, especially lentil (Khazaei et al., 2019). Usually, lentil is sown in autumn or early winter in South Asian countries and Mediterranean environments, regions that face intermittent drought at the vegetative stage and regular drought throughout the reproductive period (Mishra et al., 2018). Lentil is highly sensitive to drought at seedling and flowering stages (Shrestha et al., 2006a; Muscolo et al., 2015), and it is moderately sensitive at flowering and pod formation stages (Shrestha et al., 2006b; Mishra et al., 2016). Thus, developing drought-tolerant cultivars could potentially enhance lentil yields in drought-affected regions (Ulemale et al., 2013; Donat et al., 2016).

Lentil genotypes can be classified as drought-tolerant, drought-sensitive, or drought-adaptive based on their responses to drought stress. Drought-tolerant genotypes maintain normal functions and protect cells from dehydration through mechanisms like stomatal closure, turgor maintenance, oxidative defense, and accumulation of osmoprotective molecules (Sehgal et al., 2017; Sánchez-Gómez et al., 2019). Besides, drought-adaptive genotypes grow rapidly and complete their life cycle quickly. These genotypes exhibit greater weight of dead leaves and root length density and a decrease in photosynthesis rate, stomatal conductance, and leaf transpiration under drought conditions (Gorim and Vandenberg, 2018; Shunmugam et al., 2018). Drought-sensitive genotypes suffer from osmotic and oxidative stresses, lacking the necessary adaptation mechanisms to combat drought. These genotypes may activate their antioxidant defense system but have limited ability to adjust metabolism and handle the over-production of reactive oxygen species (ROS) (Öktem et al., 2008).

Developing drought-tolerant variants is a top priority for domestic and global lentil breeding initiatives (Erskine et al., 2011). Understanding the physiological, biological, and molecular responses of lentil to water scarcity is crucial in regard to this process. Despite various studies have investigated the responses of lentil to drought stress, there is still a lack of a comprehensive understanding of lentil adaptation to drought at morpho-physiological, biochemical, and molecular levels. This gap hinders the development of coordinated approaches, especially those using omics-based strategies, to create drought-tolerant cultivars. Furthermore, numerous investigations have been conducted to pinpoint the functional genes implicated in lentil’s ability to withstand stress, employing various techniques such as transcriptomics, QTL (quantitative trait loci) analysis, and GWAS (genome-wide association study) (Cormier et al., 2014; Swamy et al., 2018). This review provides a comprehensive discussion on the morpho-physiological, molecular, and adaptative responses of lentil in response to water-shortage conditions. It also highlights how omics-assisted breeding approaches can be utilized to develop lentil varieties that are tolerant to drought stress.

## 2 Morpho-physiological adaptation of lentil to drought stress

### 2.1 Morphological adaptations

#### 2.1.1 How do root traits and root architecture play roles in drought adaptation of lentil?

Several studies showed significant differences in root traits, such as root length, root diameter, root hairs, root area, and root volume, between tolerant and sensitive lentil genotypes under drought conditions (**Table 1**).

**Table 1:** Role of root system architecture in lentil drought adaptation.

Genotypes	Root related traits	Mechanism of tolerance	References
IC560051	Total volume	Adapted to drought by restricting the reduction of total root volume	(Priya et al., 2021)
IC559665	Total length and projected area	Extension of root length and area to forage more water	
IC248693 IC208336 IC559907	Average diameter, thickness, and tip number	Thickening of the root cell wall by absorbing food materials and maintenance of greater tip numbers	
DPL 53, JL 1, and IPL 98/193	Length and dry weight	Higher biomass and dry matter after drought spells and more extended root length help to reach the rhizosphere	(Kumar et al., 2012)
*L. odemensis* IG 72623	Biomass, total number of tips, average diameter, and total length	Wild genotypes like *L. odemensis* IG 72623 had significantly lower root biomass, total number of tips, average diameter, and total length reduction compared with cultivated lentil genotypes	(Rabani, 2018)
RIL population (ILL6002 × ILL5888	Rooting system	Robust rooting system to overcome drought effects	(Saha et al., 2010)

**Figure 1:**
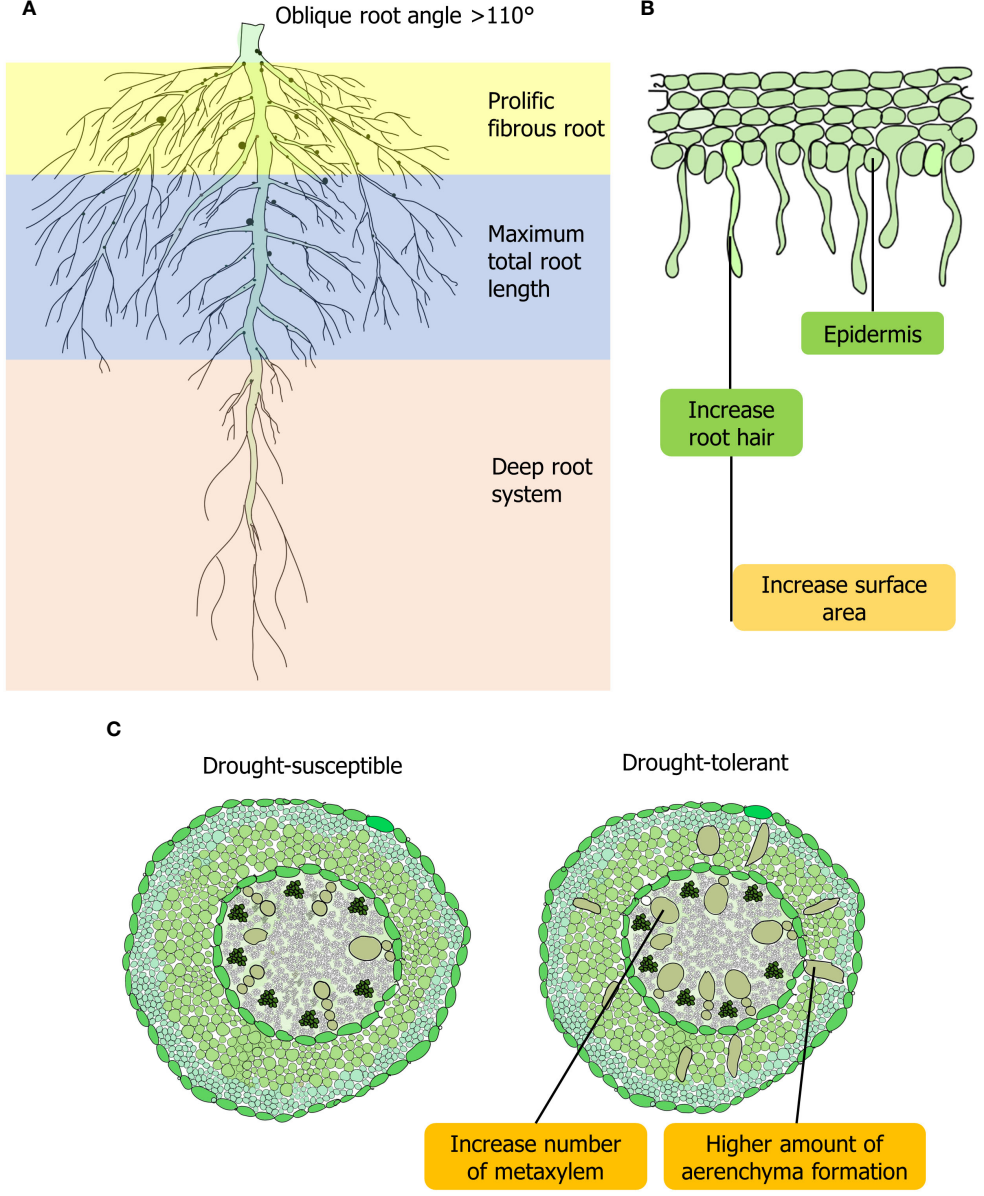
Root feature of lentil for adaptation to drought stress. **(A)** Morphological and anatomical adaptations to drought in lentil involve prolific lateral root growth, penetration into lower soil horizons, and increased root volume in drought-tolerant varieties. **(B)** Dense root hairs contribute to the enhanced root volume in lentil. **(C)** Drought-tolerant lentil may exhibit higher aerenchyma formation in their roots, similar to other leguminous crops, in response to drought stress.

In drought conditions, tolerant lentil have better water-extracting abilities from the lower soil horizon through longer roots (Kumar et al., 2012). The drought-tolerant lentil genotype has a profuse fibrous root system with maximum total root length (TRL) (**Figure 1A**). In susceptible lentil genotypes, root diameter was less than 2 mm, whereas, in tolerant genotypes, diameter ranges between 2 and 5 mm, which enables roots to uptake essential nutrients from far distance effectively (Gorim and Vandenberg, 2017). Wild lentil genotypes exhibit higher TRL with different root diameters allocated to deeper soil layers, which increases the efficiency of water and nutrient absorption from the soil (Gorim and Vandenberg, 2017). In contrast, TRL and the proportion of root length below 30 cm are not always beneficial for lentil in a semi-arid environment (Bourgault et al., 2022). Root hairs increase the total area of roots and compensate for lower root enlargement caused by drought in lentil (Subbarao et al., 1995) (**Figure 1B**). In searching for water in the soil, root hairs assist in connecting with soil particles and colloids. A significant correlation between root hair traits (hair length and density) and nutrient uptake of lentil genotypes was observed (Wasson et al., 2012; Rabani, 2018). Root hair is the outgrowth of a single rhizodermal cell. During drought, rhizodermal cells and trichoblasts increase plant hydrotropism and play a significant role in the plant’s search for and absorption of water and nutrients in different plants, including lentil (Jaffe et al., 1985; Takahashi et al., 2003) (**Figure 1**). Plant microRNA MiR-393 influences development of lateral root volume. MiR393 can promote root-mediated drought tolerance by triggering growth hormone signaling, especially in root hairs of droughts escaping legume crops (Hussain et al., 2015). Drought-tolerant lentil genotypes have a greater proportion of root volume that increases root penetration into the lower soil horizon (Gorim and Vandenberg, 2017) (**Figures 1A, B**). Lateral root volume at approximately 90% of the overall length is the major portion of overall root length in plants (Pierret et al., 2006; Zobel et al., 2007). Higher lateral root volume is considered essential because this trait is linked to a plant’s enhanced growth in a water-shortage environment (Idrissi et al., 2015) (**Table 1**). Drought-tolerant genotypes of lentil and wheat possess improved root volume that can be characterized by the early root and shoot vigor under drought conditions (Rabani, 2018; Hendriks et al., 2022) (**Figure 1B**). By lowering evaporative loss, aboveground early vigor can also aid in the retention of soil moisture under water-deprived conditions (Gorim and Vandenberg, 2017).

Root angle, a distinguishing drought-adaptive trait, controls the horizontal and vertical arrangement of roots into the rhizosphere. An oblique root angle found in lentil helps to save energy while penetrating at a greater depth of soil, and soil moisture can be utilized effectively under drought (Wendel et al., 2022; Zhang et al., 2024) (**Figure 1**). Deep roots are ideal for terminal drought conditions observed in different lentil genotypes and a perfect root system for intermittent drought would be shallow and extensive to capture sporadic rainfall effectively (Rao et al., 2024) (**Figure 1**). Different root anatomical structures, such as metaxylem, xylem vessel, and cortical play a vital role for drought tolerance in lentil. The smaller outer cortical cells support and protect the root from deformation, which also aids in soil penetration. A higher abundance of metaxylems and aerenchyma production correlates positively with drought tolerance in soybean (*Glycine max*), a leguminous crops like lentil (Prince et al., 2017). This formation of root cortical aerenchyma or reduction in living tissue area conserves energy and facilitates improved soil penetration and exploration, thereby aiding in drought adaptation (Karlova et al., 2021) (**Figure 1C**). This phenomenon of increased aerenchyma formation under drought conditions has not yet been validated in lentil, warranting further investigation. Structural arrangement and reorganization of xylem play a crucial role in drought tolerance in lentil (Purushothaman et al., 2013). Axial pressure on roots and water-use-efficiency are also influenced by the diameter of xylem vessel in legumes (Lynch et al., 2014; Prince et al., 2017). Additionally, xylem diameter was shown to be a more heritable trait than the number of seminal root axes (Richards and Passioura, 1981). Furthermore, genotypes having extensive cortical exhibit enhanced root development and water absorption, deeper root systems, improved stomatal conductance, and leaf CO_2_ assimilation to counteract drought effects.

Root system architecture (RSA), expressed as the size, shape, and proliferation of the root system, plays a crucial role in plant acclimation under moisture deficit conditions (Manschadi et al., 2008). An extensive and robust root structure and higher biomass fraction are needed to absorb nutrient and water resources far from the plant’s origin (Kou et al., 2022). RSA expresses plasticity to diversified weather conditions by enhancing the total amount of fibrous roots and decreasing lateral root area (Meister et al., 2014; Ranjan et al., 2022). The significance of root-associated parameters for drought tolerance is an increasing concern for breeders, but generally, because of the pleiotropic effect and less broad sense heritability, these parameters have been underestimated (Malamy, 2005). Phenotyping for root difference is troublesome among the large number of segregating lentil populations. Until now, few studies on drought-adaptive root anatomical traits in lentil have been done. In drought-tolerant breeding programs, maker-assisted selection for root traits should be prioritized (Francia et al., 2005). Further research in RSA will improve phenotypic efficiency and accelerate the discovery of additional anatomical traits important for drought resilience. Phenotyping of root anatomical traits can play a vital role in drought-adaptive lentil line selection.

Substantial progress in omics approaches has expanded the knowledge of drought-related genes and other regulatory elements as well as the comprehension of how RSA is remodeled at the molecular level. QTLs responsible for the RSA regulation would be extremely useful in lentil breeding programs aimed at developing drought-tolerant varieties. To establish a vigorous RSA, high importance should be given to exploring functions of different QTLs, signaling components, transcription factors, microRNAs, and their interactions with plant hormones that control root morphology and anatomy.

#### 2.1.2 How do leaf-related traits play roles in drought adaptation of lentil?

**Figure 2:**
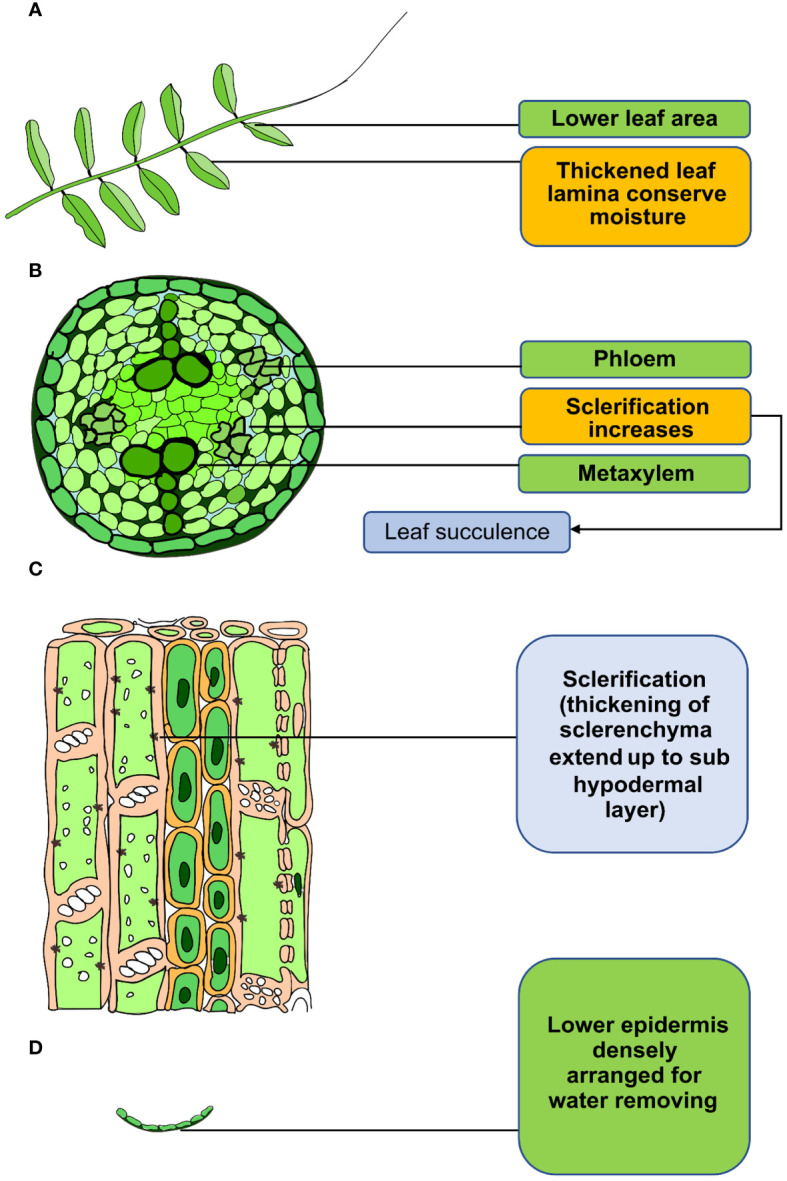
Adaptation of lentil stem and leaf anatomical structure in response to drought. **(A)** Thicken leaf lamina and pubescence reduce water loss. **(B, C)** Sclerification of the xylem reduces water loss, resulting in leaf succulence. **(D)** Dense epidermal cell arrangement in the lower epidermis reduces water loss from lentil.

Morphological modifications in different lentil genotypes occur to avoid and escape drought, including leaf orientation, phyllotaxy, and leaf surface characteristics, such as hairs (pubescence). Higher pubescent consistency in leaves can slow transpiration and conserve moisture shortage in lentil (**Figure 2**) (Talukdar, 2013). Pubescence is also seen in leaves, stems, buds, and pods in lentil (Hoque et al., 2002). Typically, the highest level of pubescence is observed on the developing tips during the vegetative phase and on the inflorescence during the reproductive phase (Shrestha et al., 2009). Leaf orientation, such as changes in leaf angle and leaf rolling, can reduce leaf temperature under terminal drought in lentil (Shrestha et al., 2009). Also, an erect leaf angle lowers the transpiration rate and preserves soil water for use later in the season (Ghanem et al., 2017). In lentil, most of the modern cultivars have more vertical leaves than the wild relatives, bringing about higher leaf area index (LAI) (Whitehead et al., 1998). In drought-stressed lentil plants, LAI is lower because leaf initiation and expansion happened later compared with drought-adaptive plants. Consequently, a reduction in average growth and an accelerated premeiotic senescence were observed (Shrestha et al., 2006a). This adverse effect in leaves can be reduced by cuticular waxy layers in leaves, stems, and flowers (Guo et al., 2016). It has been reported that cuticular waxes play a vital role in the abiotic stress-related responses in plants (Dhanyalakshmi et al., 2019). Drought-tolerant lentil genotypes exhibited greater epicuticular wax content that present on leaves than the other accessions, which plays a vital role in preventing desiccation (Ashraf et al., 1992) (**Figure 2**). Sophisticated lower epidermis protects the leaves from excess transpiration under drought condition. Tolerant lentil genotypes exhibit densely arranged phloem, as well as higher sclerenchyma tissue and greater fiber in stems and pods (**Figure 2**) (Welsh-Maddux et al., 1994).

Overall, researchers have directed their attention to lentil canopy architecture. Leaf size and light interception have received greater priority over other characteristics in lentil. Furthermore, measurements of canopy temperature and photosynthetic traits could help to identify the drought-tolerant lines. Several QTLs have been found related to shoot architecture in lentil. Due to the environmental variation and epigenetic effects, introgression of leaf adaptive traits for drought tolerance lentil breeding programs will not be effective other than validation of marker-assisted breeding.

#### 2.1.3 How does lentil adapt to drought stress during vegetative and reproductive stage?

Drought-tolerant and susceptible genotypes show different branching habits. In drought-sensitive lentil, numerous shoots give rise to greater primary and secondary branches with a higher number of podding nodes (Akter et al., 2021). On the contrary, in drought-tolerant lentil plants, the growth habit is based on one perpendicular twig with a few pods with suppression of lateral branches (Basu et al., 2022). One stem, erect/semi-erect stature, and less bushy forms are the adaptation mechanisms of lentil in arid environments (Gahoonia et al., 2005, 2006; Sarker et al., 2005). The pit membrane diameter of stems and branches exhibited lower exposure to drought and assisted in increasing horizontal water conductance. The tracheid density and pit density generally showed a decreasing trend during drought stress, which was found to lower water transport efficiency for escaping drought in lentil (Priya et al., 2021). Branching extends up to mid-vegetative phenophase and ends after the initiation of the flowering stage. In severe drought-prone areas, lentil genotypes escape terminal drought through early flowering with synchronous maturity. However, the post-anthesis stage requires enough moisture to sustain yield under drought (Kottmann et al., 2016). Some short-duration accessions of lentil that show satisfactory pod filling in normal conditions can be tested for their potential for drought avoidance (Khatun et al., 2021). Early pod setting is another principal drought-escaping strategy of some lentil cultivars. In drought-susceptible genotypes, lentil yield reduction was recorded up to 24% during a severe drought period during pod development (Sarker et al., 2005). Water scarcity severely affected biochemical processes (sugar metabolism), contributing to abnormal seed formation and pod enlargement with reduced seed size and shape, but the plant completed the reproductive stage quickly to give offspring (Shrestha et al., 2006a). There is evidence of yield superiority in the tolerant genotype of the green lentil (microsperma) compared to the red lentil (macrosperma) under water-deficit conditions (Mishra et al., 2016). The greater leaf relative water content, root/shoot ratio, pod number per plant, and seed number per pod contributed to satisfactory yield in microsperma genotype HUL-57 over the macrosperma IPL-406. However, the underlying drought-resistance mechanism in HUL-57 is not fully known (Mishra et al., 2016).

Early flowering, higher biomass production, greater branching, and podding nodes are regarded as vital characteristics to avoid drought effects on lentil. Also, these are the main criteria for identifying high-yielding drought-tolerant lentil lines. However, in most of the lentil genotypes, rapid completion of the reproductive cycle sacrificed yield. The QTLs linked to early flowering and greater biomass need to be transferred to the high-yielding parent through the backcross breeding technique.

### 2.2 Physiological adaptations

**Figure 3:**
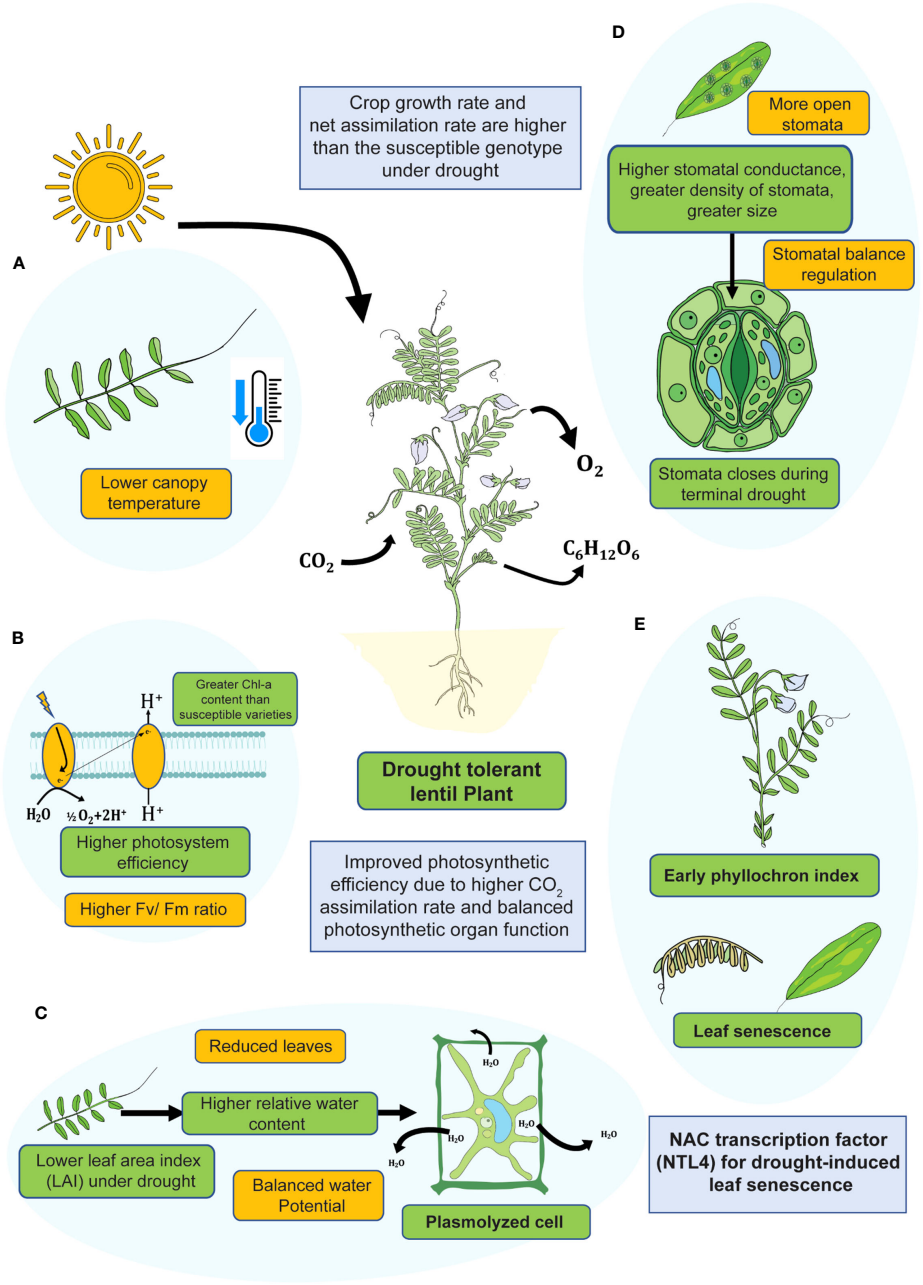
An overview of physiological adaptation strategies of lentil plant under drought stress during vegetative phase. Drought-tolerant lentil genotypes showed **(A)** lower canopy temperature, **(B)** higher photosystem efficiency, **(C)** higher relative water content, **(D)** better stomatal regulation, and **(E)** early phyllochron index.

In drought-sensitive lentil, stomatal conductance is not so robust that the photosynthetic rate is lower. Although in this case, total carbon fixation is narrow, which is not linked with the assimilation ability (Lawlor and Cornic, 2002). In contrast, drought-tolerant genotypes had a greater CO_2_ diffusion rate and a higher level of carbon fixation that positively affected pod yield (Fikiru et al., 2011) (**Figure 3**).

Relative water content (RWC) is one of the special characteristics for assessing drought tolerance in plants. In lentil, RWC decreases considerably at the onset of the vegetative stage and continues up to the reproductive stage. After 13-20 days of drought, the drought-tolerant lentil genotype had comparatively higher RWC than the drought-susceptible lentil (Talukdar, 2013) (**Table 2**). In addition, lentil plants avoid drought by developing epidermal cells, subsidiary cells, and guard cells present in the stomata and trigger gene expression related to photosynthesis under stress conditions (Chaves et al., 2009). In addition, plants can become more resilient to drought stress by blocking the synthesis of acetyl-CoA carboxylase and reducing the rate at which they respire (Gu et al., 2020). The decrease in respiration rate is typically correlated with drought resistance in plants (Yang et al., 2021). With lower root respiration and biomass production, an alternative way of lentil germplasms to survive in water-deficient soil, fewer free radicals are produced (Liu and Li, 2005; Yang et al., 2021). Alternative oxidase pathways help to balance ROS level during mitochondrial respiration (Blokhina, 2003). Under water-deficit conditions, a lower degree of stomatal opening leads to lesser activation of physiological traits in lentil (Ghanem et al., 2017). Lentil’s primary response to drought stress is ABA-mediated stomatal closure. Stomata are typically closed in order to lower the rate of transpiration, which also lowers the rate of gas exchange. Drought-tolerant genotypes displayed higher levels of chlorophyll, RWC, and biomass under drought stress than the sensitive genotypes (Biju et al., 2021) (**Figure 3**), which suggests a higher level of photosynthesis in drought-tolerant genotypes under water-scarcity. Under limited water conditions, stomata transpiration inhibition activity is decreased in South Asian lentil (Ghanem et al., 2017). Leaf senescence after the onset of drought is a physiological process, whereas adaptive genotypes of lentil make available nutrients for specific periods. During leaf senescence, plants lose their leaves, and transpiration is reduced to save water.

**Table 2:** Physiological mechanism of drought tolerance in different lentil accessions.

Accession name	Type of accessions	Physiological adaptation strategies	References
PDL-1 and PDL-2	Drought-tolerant	• Close stomata under terminal drought that brings about a lower transpiration rate • PDL-2 contained comparatively higher chlorophyll content than other genotypes	(Singh et al., 2016)
ILWL-436 and ILWL-314	Drought-tolerant wild	• Non-significant reduction of chlorophyll content and relative water content (RWC)	(Singh et al., 2016)
JL-3 and E-153	Drought-susceptible wild	• Significant reduction of RWC • Opening of stomata results in higher transpiration rate	(Singh et al., 2017)
PL-4	Drought-tolerant	• Increased stomatal density and declined the size of stomata	(Talukdar, 2013)
IPL-406	Drought-sensitive	• Early phylochron index • Lower senescence • Lower cell membrane integrity	(Mishra et al., 2018)
HUL-57	Drought-tolerant	• Late phylochron index • Higher senescence Greater cell membrane integrity	(Shrestha et al., 2006a)
GP-3643 and IC248956	Drought-tolerant	• Higher stomatal density under osmotic stress	(Sinha et al., 2018)
PBA Jumbo2	Moderately drought-tolerant	• Higher individual leaf area, radiation use efficiency, and harvest index	(Tefera et al., 2022)
Fırat 87 and Çiftçi	Drought-tolerant	• No changes in chlorophyll content and RWC under drought	(Morgil et al., 2017)
Sultan	Drought-sensitive	• Reduction in chlorophyll content and RWC in response to drought	
LL 7833, ILL 7835 & ILL 7814	Drought-tolerant	• Transpiration is reduced in response to drought	(El Haddad et al., 2021)
ILL 4605, ILL 8025 & ILL 7286	Drought-sensitive	• Transportation did not reduce in response to drought	
ILL-6002 Indianhead	Drought-tolerant	• Tolerant genotypes showed lower levels of biomass production, chlorophyll content, and RWC reduction in response to drought stress than susceptible genotypes	(Biju et al., 2021)
PI-468898 ILL-7537	Drought-sensitive		
ILL-6002	Drought-tolerant	• The tolerant genotype exhibited higher levels of net CO_2_ assimilation and internal CO_2_ concentration, along with lower levels of stomatal conductance and transpiration under drought stress compared to the sensitive genotype	(Biju et al., 2023)
ILL-7537	Drought-sensitive		

## 3 Physiological and molecular adaptation of lentil under drought stress

### 3.1 Biochemical adaptations

In drought-tolerant lentil genotypes, proline accumulation is higher than in the drought-sensitive genotypes (Sinha et al., 2018). The level of proline varies with different growth stages of lentil (Allahmoradi et al., 2013). By acting as an osmoprotectant, proline plays a vital role in protecting complex protein structures and cell membranes from drought-induced effects. Sucrose accumulation is higher in drought-tolerant genotypes than in drought-sensitive lentil varieties. When photosynthesis is downregulated under drought conditions, starch breakdown occurs to release hexose needed for survival (Bandeoğlu et al., 2004; Mishra et al., 2016). Oxidative stress indicators, including hydrogen peroxide, malondialdehyde, and methylglyoxal, accumulated at lower levels in drought-tolerant genotypes when compared with drought-susceptible genotypes. Also, drought-adaptive genotypes accumulate high levels of active solutes, metal ions, and secondary compounds in cytosol to counter osmotic stress (Shrestha et al., 2006a, b; Vadez et al., 2012). Superoxide dismutase, ascorbate peroxidase, and catalase activities showed significant up-regulation in drought-tolerant lentil genotypes in comparison with tolerant genotypes (Sarker and Oba, 2018). Under drought stress, lentil genotypes that were tolerant showed greater concentrations of total flavonoids, total phenolics, and tannins than genotypes that were sensitive (El Haddad et al., 2021). These metabolites serve as osmoprotectants and are essential in scavenging free radicals to prevent oxidative damage induced by many abiotic stresses (Bueno and Lopes, 2020). Total phenolic and flavonoid contents are potential antioxidants, and plants that accumulate them are more resilient to drought stress (Hasan et al., 2020). Different enzymatic responses were observed in drought-tolerant and drought-susceptible genotypes. Higher acid phosphatase activity is directly linked with drought tolerance in lentil. Macrosperma-type lentil exhibited lower acid phosphatase activity than microsperma, indicating genotype-dependent enzymatic action (Mishra et al., 2016). Drought-tolerant genotype L-4594, which belongs to the macosperma group, showed higher invertase activity than drought-susceptible genotype L-4076. A lower reduction (2.36%) of nitrate reductase (NR) activity was found in drought-tolerant genotype PL-2 compared to other genotypes (Eesha et al., 2022).

### 3.2 Molecular mechanism of drought adaptation

Drought tolerance has long been known to be a complicated phenomenon involving the coordinated action of numerous genes, encoding for membrane-stabilizing proteins, aquaporins, seed proteins, heat shock proteins, dehydrins, and late embryogenic abundant proteins (Umezawa et al., 2006; Skirycz et al., 2011; Claeys and Inzé, 2013). Tolerant varieties of lentil get signals through osmotic and ionic effects and membrane fluidity changes in the membrane receptors (Farooq et al., 2020). Membrane-tethered transcription factors act to induce drought stress-inducible genes in the membrane and provide a connection between stress response and developmental pathways (Slabaugh and Brandizzi, 2011). At the transcription level, lentil genotypes of *L. culinaris* exhibited prompt response under drought stress. In lentil species, Ca^2+^-dependent protein kinases (CDPKs) were significantly up-regulated under drought and heat stresses (Hosseini et al., 2021). Besides, *succinate dehydrogenase flavoprotein subunit 1*, namely (*SDH1-1*) located in mitochondrial respiratory chain complex-II was found up-regulated in lentil under drought stress (Singh et al., 2017). Myeloblastosis (MYB)-like protein and *Mt*bZIP124 transcription factor act as a positive regulator of drought tolerance in lentil by upregulating expression profiles of many drought-responsive genes under drought stress. In contrast, acid phosphatase VSP1, acts as a negative regulator in the ABA signaling pathway and is down-regulated in the drought-tolerant lentil genotype (Singh et al., 2017). Many protein families play a strong influence on the survival of lentil plants under drought circumstances. Among them, the aldehyde dehydrogenase (ALDH) family’s proteins have a substantial relation with the removal of aldehyde that is toxic to plants and was greatly up-regulated in lentil grown under drought conditions (Singh et al., 2017). Moreover, lysine related demethylase gene (*JMJ-30*) has a connection with early flowering to escape terminal drought (Jones et al., 2010). *Glutathione peroxidase* gene has a strong influence in mitigating drought stress in lentil by upregulating the detoxification of ROS. In lentil, the *Galactinol synthase 1* gene was found many folds up-regulated and has the function to mitigate drought effects. Through gene expression study, it is obvious that the gene liable to leaf senescence and lower stomatal conductance was up-regulated highly in tolerant lentil genotypes than the sensitive ones (Hosseini et al., 2021). Diacylglycerol acetyltransferase WSD1, responsible for wax formation, play pivotal roles in counteracting heat and drought stresses (Singh et al., 2018). Lentil ascorbate peroxidase enzyme has functions in ROS detoxification through converting H_2_O_2_ into H_2_O (Singh et al., 2017). Tolerance at the molecular level is strongly controlled by proteins that help to attach DNA to the promoter area and kinase that specifically alters other proteins like mitogen-activated protein kinase (MAPK) (Joshi et al., 2016). The molecular interaction of the signaling pathway of phytohormones and miRNA is one of the drought-escaping strategies of lentil (Araújo et al., 2015; Ahmad et al., 2022).

## 4 Recent progress in lentil screening and breeding for drought-stress tolerance

### 4.1 Conventional breeding

High-yielding cultivars that are suited to various environmental conditions, including drought, have been developed with the use of conventional breeding approaches in various countries of the world (Idrissi et al., 2015; **Table 3**). For example, improved lentil varieties with excellent yield stability and adaptability to various agro-environments of Morocco have been released (reviewed in Zeroual et al., 2022). Although conventional breeding has significantly improved the genetic makeup of lentil, output has stagnated recently. Therefore, traditional breeding methods have been combined with genomics-assisted and molecular breeding technologies to accelerate the progress of breeding for developing high-yielding, drought-tolerant lentil varieties (described in more detail in section 4.2).

**Table 3:** List of drought-tolerant lentil varieties developed through conventional breeding approaches.

Genotypes/Accession	Country of origin	Adaptation Strategies	Variety/line	Year of release	Pedigree	References
Beredu	Ethiopia	Drought-tolerant	Variety	2019	FLIP2911-17L (ILL814 XI LL590), 95S 35151-0	(Damte and Tafes, 2023)
L4729	India	Drought-tolerant	Variety	2019	SKL 259 X L 4147	(Asif et al., 2023)
Jemmat Shaim	Morocco	Drought-tolerant	Variety	2019	ILL4605 x ILL1005, 20005S-100-3	(Idrissi et al., 2020)
Krib	Tunishia	Drought-tolerant	Variety	2019	ILL590 X ILL8113, FLIP2012-196L, 06S54140-02	(Ouji et al., 2023)
Idlib-3	Syria	Drought-tolerant	Variety	2002	ILL99 X ILL5588	(El-Ashkar et al., 2004)
Binamasur-10	Bangladesh	Drought-tolerant	Variety	2016	Exotic germplasm of ICARDA	(Roy et al., 2019)
Kamande	Kenya	Drought- tolerant	Variety	–	–	(Njeru et al., 2021)
FLIP-96-51, IG-109039, ILL 6002, ILL 76037, ILL-9916, ILL-10893, PDL-1, PDL-2 and ILL-10893	ICARDA	Drought-tolerant	Line	–	–	(Singh et al., 2016)
ILWL-314, ILWL-437, ILWL-462	Turkey	Drought-tolerant	Wild line	–	–	(Singh et al., 2018)
ILWL-55(2)	Israel	Drought-tolerant	Wild line	–	–	(Singh et al., 2016)
ILL 6024, ILL 7618, ILL 7981, ILL 8095, ILL 8138, ILL 8621, 9830, ILL 9844, ILL 9850, ILL 9920, ILL 6024, ILL 9921, ILL 9922 and ILL 9923, ILWL-436 and ILWL-314	ICARDA	Drought escaping	Line			(Singh et al., 2016)

#### 4.1.1 Germplasm evaluation and cross breeding

Numerous wild and cultivated lentil germplasm have been screened to assess the variability among root and shoot traits for the selection of parents for developing drought-tolerant varieties (Gorim and Vandenberg, 2017). Due to several factors, including high genotype-environment interactions, unknown wild germplasm lines, lack of reliable information for particular traits, and associated linkage drag, plant breeders are reluctant to use new germplasm resources for drought screening (Kumar et al., 2013). Screening of lentil has been conducted targeting morphological and physiological parameters in both control and drought conditions (Tahir et al., 2021). A reproducible protocol for phenotyping of drought stress tolerance was established employing a hydroponic system to select the best line tolerant to drought stress (Singh et al., 2013). Breeders have emphasized deep rooting and high biomass yield contributing lines confirmed through morphological phenotyping for drought adaptive variety development in ICARDA (arid region of India) (Buddenhagen and Richards, 1988). Drought-tolerant lentil varieties have been developed through hybridization (Roy et al., 2019; Ouji et al., 2023). *Berudu*, *Extra*, *Krib*, and *Kamande* are drought-tolerant lentil varieties developed in different parts of the world, including Ethiopia, Morocco, Tunisia, and Kenya (**Table 3**). The common cultivars *ILL6002* (India) and *Binamashur-10* (Bangladesh) have been used as drought-tolerant checks in many breeding programs released by national organizations (Sarker et al., 2005). Different lentil species exhibit different levels of drought tolerance. However, varieties developed through cross-breeding approaches in lentil possess higher levels of tolerance (Hamdi et al., 1996).

#### 4.1.2 Mutation breeding

**Figure 4:**
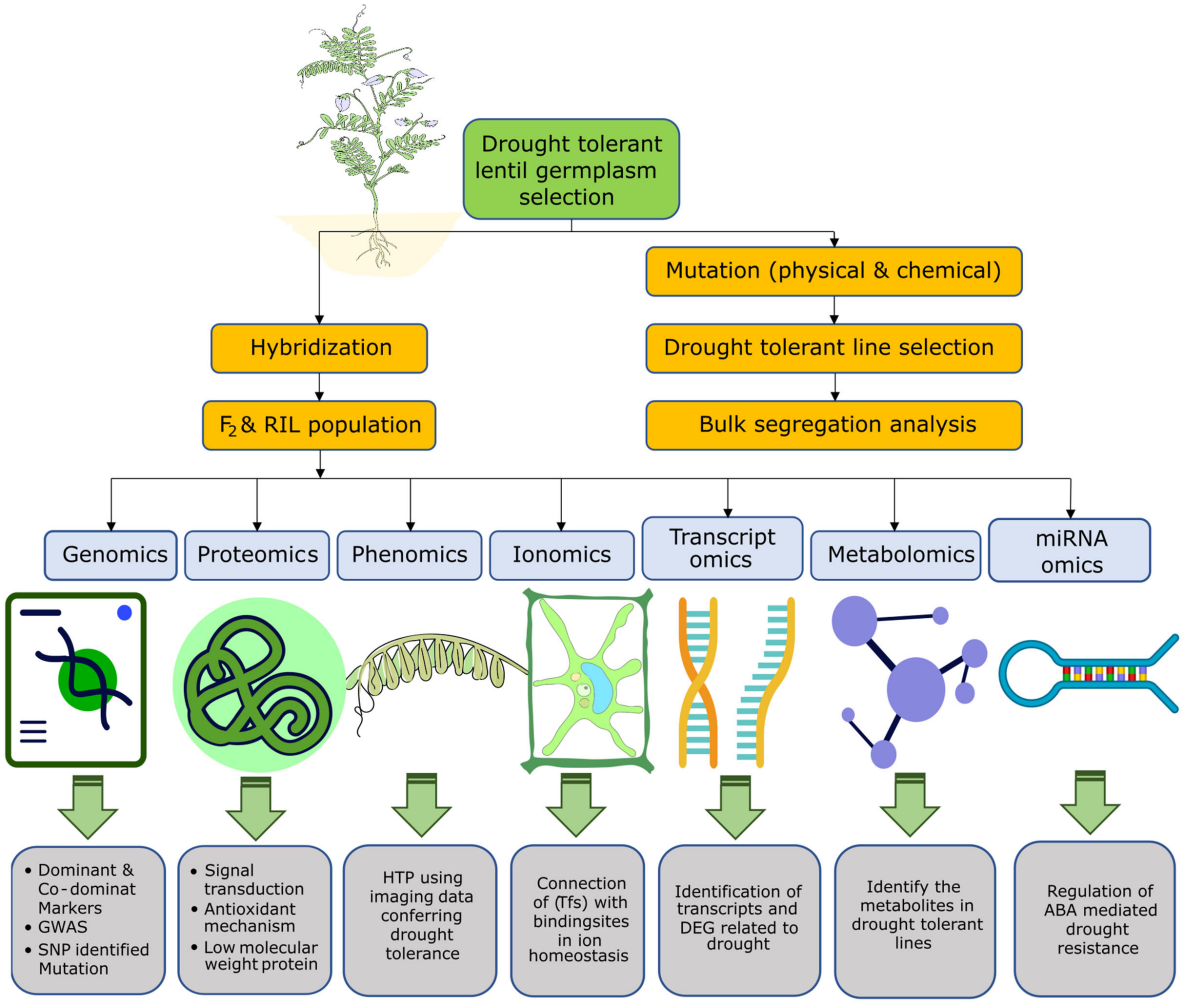
Overview of the omics-based techniques and their use in drought-tolerant lentil breeding. Genomics, proteomics, phenomics, ionomics, transcriptomics, metabolomics, and miRNA omics approaches were used individually or in combination to dissect the response of lentil under drought stress and screen or breed drought-tolerant lentil.

Low seed sets in inter-specific hybridization, minuscule flower size, floral drop, and lack of well-established embryo rescue techniques restrict the further use of hybridization for lentil improvement (Roy et al., 2022). Within these intrinsic limitations, induced mutation breeding offers a cogent strategy for augmenting the genetic variability of lentil by widening the genetic base. Advanced mutant lines can be developed with desired traits of interest via the application of mutagenic agents. Researchers have used physical (gamma ray) or chemical mutagens (ethyl methane sulphonate and sodium azide) to induce genetic variability in lentil for developing moisture deficit or drought-tolerant mutants (Tomer et al., 2007; Ali et al., 2010; Darai et al., 2016; Tabti et al., 2018; Roy et al., 2022). For example, a few advanced mutants of lentil tolerant to drought stress have been identified by Tomer et al. (2007). A drought-tolerant mutant line, *AEL 23/40* released in Pakistan developed through the application of 100-600 Gy in the cultivar *ICARDA-8* (Ali et al., 2010) (**Figure 4**).

### 4.2 Omics-based approach

The overview of the implications of different omics-based techniques in drought-tolerant lentil breeding is presented in **Figure 4**.

#### 4.2.1 Genomics

In a study, 133 dominant or co-dominant markers were used for root- and shoot-related traits in lentil (Idrissi et al., 2015). In another study, 495 SSR (simple sequence repeat) primers, 35 showed polymorphism, where 278 genotypes were used for drought-tolerant genotypes screening (Singh et al., 2016). In addition, excessive and dominant allele-recognizing markers and competitive allele-specific PCR have been widely used to screen drought-tolerant genotypes (Sharpe et al., 2013; Kaur et al., 2014). Tolerance to drought stress is linked with numerous genes. Single nucleotide polymorphism (SNPs), an advanced molecular marker, has become widely used because of the lack of closely linked markers and the creation of higher-resolution linkage maps. The use of GWAS to find SNPs that indicate drought tolerance in lentil has grown in popularity (**Figure 4**). A candidate gene interference study can be conducted with either gene chips or qPCR (quantitative polymerase chain reaction) for observing stomatal activity and also associated candidate genes involved (Reddy et al., 2004; Shinozaki and Yamaguchi-Shinozaki, 2006; Talame et al., 2006; Guo et al., 2009; Kim et al., 2010; Anjum et al., 2011; Joshi et al., 2016; Fàbregas and Fernie, 2019).

#### 4.2.2 Proteomics

Proteomics unveils the functions of various drought-responsive proteins involved in signal transduction, redox homeostasis, and cellular defense. These proteins are crucial in safeguarding and adapting plants to adverse environmental conditions, including drought (Jha et al., 2022). Proteomic analysis was conducted on different pulse crops such as chickpea (*Cicer arietinum*) (Heidarvand and Maali-Amiri, 2013), pigeon pea (*Cajanus cajan*) (Krishnan et al., 2017), pea (*Pisum sativum*) (Jan et al., 2023), and common bean (*Phaseolus vulgaris*) (Wang et al., 2017). In general, these proteomic analyses showed how abiotic stresses altered the expression of specific proteins. Several previous reports demonstrated the proteome profile of seeds of different lentil genotypes (Scippa et al., 2008, 2010; Shaheen et al., 2019). However, limited information is available on the proteomic changes of lentil in response to drought stress. Thus, the proteomic response of lentil accessions will open up the possibility of investigating the mechanisms of drought tolerance in lentil (Jan et al., 2023). Further studies should be conducted to explore the molecular changes, interactions, post-translational modifications, signaling roles, and subcellular localization of major proteins that are liable to moisture-deficit tolerance.

#### 4.2.3 Phenomics

Phenomics plays a crucial role in detecting alterations in plant phenotypes caused by different abiotic stresses, including drought. Surprisingly, no research has been conducted that utilized phenomic techniques, such as high throughput phenotyping (HTP), to identify drought-induced morphological changes in lentil (**Figure 4**). In HTP technology, imaging data can be collected and analyzed using a robust screening protocol. Plant temperature, nutrient content, and photosynthetic efficiency can be calculated using near-infrared spectroscopy. Other physiological attributes, such as water use efficiency and rate of transpiration, may easily be calculated in standing crops using HTP. Phenotyping of lentil can face obstacles due to the same phenological structure as other crops, and environmental factors can influence the accuracy. Therefore, robotic measurement with an accurate navigation system is the best way of collecting morpho-physiological data on lentil (Salas Fernandez et al., 2017).

#### 4.2.4 Ionomics

Ionomics assists in comprehending the role of the plant ionome during stress and in identifying the genes and regulatory pathways related to mineral accumulation, translocation, and participation in various molecular mechanisms under both non-stress and stress conditions. Drought specifically down-regulates mineral nutrition. Nutrient uptake of lentil was affected in the early stage due to drought. In lentil, iron, zinc, and manganese uptake is severely hampered under drought stress (Malik et al., 2022; Zeroual et al., 2022). The lentil genotypes that demonstrated higher mineral uptake are better adaptive to drought conditions. The changes in the ionome of lentil in response to drought stress have not been studied yet. The ionome of different lentil landraces or genotypes should be analyzed to identify lines with superior nutrient homeostasis capabilities under drought stress. These genotypes can be utilized in future breeding programs (**Figure 4**).

#### 4.2.5 Transcriptomics

Transcriptomics identifies differentially expressed genes, transcripts, and regulatory networks that control plant responses to drought (**Figure 4**). A sum of 6633 differentially expressed genes (DEGs) was identified and confirmed through the Illumina HiSeq 2500 platform. Those genes are involved in drought tolerance by regulating carbon and amino acid metabolism and plant hormone signal transduction (Singh et al., 2017). A total of 9949 SSRs, 8260 SNPs, and 1248 INDELs markers were used in the transcriptome profiling of lentil for drought tolerance screening and this approach will be helpful for further functional studies for transcripts (Singh et al., 2017). A sum of 18,369 transcripts were identified and traced for drought tolerance in lentil; among them, up-regulation took place in 11435 transcripts, and down-regulation took place in 6934 transcripts. The study also revealed that lower conductance in stomata is significantly up-regulated in drought-adaptive germplasm (Singh et al., 2017). Furthermore, in short-term drought, 6949 DEGs and 2915 DEGs were found in the leaf and root, respectively; in long-term drought, 8306 DEGs and 18327 DEGs were found in the leaf and root for long-term drought. In response to extended dry spells, genes linked to protein ubiquitination, seed and cell wall development, and transcription activities were up-regulated in roots, while genes linked to osmotic stress, abscisic acid, and other related processes were down-regulated in leaves. Additionally, genes related to the tyrosine kinase signaling pathway, circadian rhythm, chloroplast organization, and other related processes were down-regulated (Zeroual et al., 2022).

#### 4.2.6 Metabolomics

Metabolomics facilitates the study of metabolites linked to plant adaptation to abiotic stress (Malik et al., 2022). Significant alterations in several metabolites linked to critical cellular metabolic processes, such as protein metabolism, glycolysis, TCA cycle, carbohydrate metabolism, and several hormonal homeostasis, were studied under drought stress (Jha et al., 2022). According to Zeroual et al. (2022), primary metabolites, including glucose, sucrose, and trehalose, act as signaling molecules to control the expression of genes related to plant growth and the stress response. The metabolome of drought-tolerant cultivar Elpida and drought-sensitive cultivar Flip03-24L were analyzed to identify potential biomarkers for screening tolerant genotypes during early growth stages. Variations in the accumulation of specific metabolites, including D-fructose, α-trehalose, myo-inositol, and L-tryptophan, were observed between the two contrasting genotypes. This study provides insights into different aspects of lentil metabolism under drought conditions. It suggests the potential for using this information to effectively identify drought-tolerant lentil germplasm in the early stages of growth (Foti et al., 2021). Higher trehalose accumulation is strongly linked with protecting cellular structure from osmotic damage (Muscolo et al., 2015). Besides, in different drought-tolerant genotypes, higher metabolite (protein, proline, and carbohydrate) content was found (Eesha et al., 2022). Also, thirty-one common metabolites were identified in both drought and salinity studies; among them polyamines, organic acids, sugars and polyols, and amino acids are common (Tiwari et al., 2022). Genomic mapping of metabolites using metabolite-based QTL mapping (mQTL) and GWAS (mGWAS) is widely used; however, not a single study has been conducted in lentil to date (Litvinov et al., 2021). Therefore, metabolomics-assisted breeding connecting QTL mapping will be helpful for efficient screening and selection of breeding material for improving yield and drought tolerance in lentil (Kumar et al., 2021).

#### 4.2.7 miRNA-omics

Non-coding RNA molecules, called microRNAs (miRNAs), control gene expression and are widely utilized in demonstrating plant tolerance attributes to abiotic stresses such as drought. Due to their enormous impact on gene expression, miRNAs have a variety of roles in molecular, biochemical, and physiological processes related to the drought response. For instance, miRNAs are associated with the regulation of both ABA- and non-ABA-mediated pathways involved in drought resistance in lentil (Leitão et al., 2020) (**Figure 4**). They may regulate stress responses, auxin responses, protein phosphorylation, ATP synthesis, and enzyme activation involved in plant growth and metabolism (Singroha et al., 2021). While miRNAs in plants can only be found through time-consuming and costly experimental methods, comparative genomics combined with innovative bioinformatic tools pave the way for fast and affordable miRNA identification using homologous sequence searches with previously identified miRNAs. An *in silico* technique was used to identify 12 novel miRNAs from 10,190 Expressed Sequence Tag, subsequences of lentil, with 73 putative targets grouped into seven miRNA families (Lakhani and Kalaria, 2018). These miRNAs may play functions in the regulation of signal transduction pathways and gene transcription in lentil.

### 4.3 Transgenic approach

The transgenic approach is one of the viable options for developing drought-tolerant lentil varieties (Gupta et al., 2020). In lentil, genetic transformation through *Agrobacterium tumefaciens* for dehydration-responsive element binding (*DREB1A*) gene introgression was studied (Khatib et al., 2011). However, extensive research to identify drought tolerance genes in lentil has not been done. Identification of stress-mitigating proteins, enzymes, antioxidants, plant hormones, and DEGs is the key to developing drought-tolerant lines in lentil (Ashraf, 2010). A holistic approach is needed for the development of drought-tolerant lines that can contribute to sustainable yield in variable drought environments. Nevertheless, developing transgenic plants is subject to numerous laws and guidelines. Before using this strategy, other related concerns, including public demand for transgenic variety in a specific location and human health risks, should be taken into account (Bawa and Anilakumar, 2013). Many countries do not allow the importation of food produced through gene transfer technologies, typically called Genetically Modified Organisms (GMO). Recently, genome editing technologies have drawn significant attention to the researchers working in this field due to the lack of barriers to variety release in most countries.

## 5 Conclusion and future prospects

Drought stress has been considered the major obstacle for lentil to obtain a satisfactory yield, especially in drought-prone areas. Low production affects the total pulse consumption, which brings about a deficit in plant protein in the daily food menu. Morpho-physiological adaptation to drought confers an improved root system, better water utilization strategy, relocation of stored energy, drought-responsive stomatal movement, and lower respiration. Achieving drought tolerance in lentil is still cumbersome due to the involvement of multiple genes in drought responses and tolerance. Transferring drought-tolerant specific morpho-physiological characters through crossing should get the best priorities to develop better drought-tolerant lentil. Genomics tools have become increasingly essential in traditional breeding methods, enabling genetic enhancement for climate-smart pulses. Transcriptomics and metabolomics have revealed noteworthy discoveries regarding the genes and metabolites associated with drought acclimation in lentil. However, additional research is required to comprehend their potential to generate drought-tolerant lentil genotypes fully. High-throughput phenotyping platforms have emerged as dependable instruments for swiftly and accurately capturing crucial phenotypic characteristics that contribute to plant adaptation to drought. Phenotyping is the main bottleneck for identifying traits that impart drought tolerance because inaccurate and imprecise phenotyping impedes genotyping progress. Consequently, the combination of omics technologies will provide useful data that may facilitate high-throughput trait dissection associated with high-yield lentil crops. Moreover, genetic engineering and marker-assisted breeding techniques can be used to target specific drought-resistant features in lentil. Combining rapid breeding methods with additional omics technologies in dry and semi-arid regions could expedite cultivar development and boost lentil yield. Future research should consider the following issues:

(i) Improvement of root and shoot architecture of lentil favorable for drought adaptation.
(ii) Identical gene-based markers should be identified to improve drought tolerance in lentil.
(iii) Identification of superior lentil germplasms with heightened drought tolerance phenotypes suitable across different drought-prone areas.
(iv) Drought tolerance breeding in lentil should consider variation in climatic conditions and the multigenic origin of the adaptive responses of lentil.
(v) Figuring out the potential of existing drought-tolerant varieties that provide satisfactory yield under severe drought conditions.
